# Mirror, mirror, on the wall, will my submission say it all?

**DOI:** 10.2349/biij.3.1.e1

**Published:** 2007-01-01

**Authors:** BJJ Abdullah, KH Ng

**Affiliations:** Department of Biomedical Imaging, Faculty of Medicine, University of Malaya, Kuala Lumpur, Malaysia

Now there was this queen who was the most beautiful woman in the whole land, and she was very proud of her beauty. She stood in front of her mirror every morning, and asked:

*Mirror, mirror, on the wall,*

*Who in this land is fairest of all?*

And the mirror always said:

*You, my queen, are fairest of all.*

And then she knew for certain that no one in the world was more beautiful than she.

˜ ˜ ˜ ˜ ˜ ˜ ˜ ˜ ˜ ˜ ˜ ˜ ˜ ˜ ˜

But the mirror also said:

*You, my queen, are fair; it’s true.*

*But Little Snow-White is still*

*A thousand times fairer than you [1]*

We do this all the time don’t we? Looking for recognition of our abilities, be it beauty, brawn or brains. Be it friendship, love, music, or writing, we constantly hound our mirror with the same question: “Am I the best?” And does this obsession extend to something as professional as getting a research paper published? Yes, it does.

The process of publishing a research paper begins right from the time you conceive the idea. And this includes all your efforts in polishing the data, reviewing available literature, writing the proposal, seeking peer reviews, hunting for funding, and preparing presentations to market your proposal. Not to mention all those sacrifices you made, that movie you never went to or that golf game you gave up. A research paper is a major investment of time, energy, and effort.

No wonder then, that it is a pretty bad scene out there, with still-born ideas stolen from previously published journals, data left un-analyzed for reasons unknown and worse still, analyses that didn’t yield the expected results. Most of the novices in this game are bowled out in the first over with the “R” word. Rejection is part and parcel of Publishing but how many of us accept the fact that our work may at some point, go into the “Rejection Pile”? Half-complete papers because of half-baked information, non-cooperative co-authors, sudden lack of inspiration to write, these are just some self-created reasons for your manuscript to reach the R pile. And like the proverbial “mirror on the wall always calls the queen the fairest of them all”, we want to see those lovely words “Paper accepted” etched on our manuscript.

How does one cope with rejection? What must you do to keep your spirit and enthusiasm up and high to ensure that all your efforts, time, money, and sacrifice is not wasted? The caveat is that all the processes in the research study were sound in the first place; the Science was definitely great; but the Art needed and still needs help; the art of how to write, how to deal with the dejection of reviewers and comments from editors. And this is an Art we all need to study and develop.

Rejections are a reality. **Your submissions will be rejected!** Say it and accept it. Out of the 100 papers that are submitted at least 50% get rejected. Sometimes even more, and if you are real lucky, sometimes less. We have forgotten that we are not the only ones that suffer rejection in scientific writing. Rejection exists in every sphere of our lives.

The best way to avoid rejection is to ensure that your research submission is of the highest standard. Make sure that the format is correct, grammatical errors are minimal, spell checks are accurate, and that the research and statistical methodology is correct. These are the simple mistakes that put off reviewers when they receive a manuscript for editing. For those who are “grammatically challenged”, it is wiser to get a professional copy editor look through your work before submitting. Sorry, but colleagues and friends are not of much use here; a professional is a professional.

Rejections hurt. And they hurt like hell! Anyone who tells you not to take this personally has probably never faced a rejection. How can it not be **personal!** Having said this, the trick is to accept it, take it in your stride, and move on. Towards pulling your work out of that damned R pile. If you are devastated by the rejection letter and feel that your heart has been drilled, please wallow in self-pity, be sad, be mad, be angry, scream and do whatever it takes to cope with it. But for God’s sakes, cope with it. If the blues persist, make excuses for the rejection. Curse the reviewers, the editors, and even the publishing house if you have to. Dish out whatever excuses you can to make yourself feel better.

One truth that we can never run away from is that **rejection** is an additional **stress** (as if we don’t have enough already!). Face this stress like you would any other. But avoid writing to the editors at this stage as you would probably live to regret the things you say. Share your sorrow about the rejection with your co-authors and colleagues (women more likely than men) as this does help lessen the hurt. Having a supportive spouse, colleague, or friend acts like an elixir and certainly does help reduce the feeling of despair or self-worth but then again, not totally.

Indulge in self-pity, but learn to put a full stop. Bouncing back is essential to success. Many a researcher has been stifled early in their career due to a poorly written rejection letter that was badly-timed, just perfect to puncture a fragile ego unused to receiving criticisms.

If you get past this stage then maybe there is hope for you, otherwise you are doomed to meet the fate that has befallen the others who chose never to write again. Remember that everyone who is at the pinnacle of success today, had to face rejection at some point in their writing career. What differentiates them from the losers is that they picked up their manuscripts from the trash and began all over again, never giving up their creativity to a worthless bin holding trash. Did you know that the first Harry Potter book is reported to have been rejected by 14 publishers? Stephen King had over 30 rejections for “Carrie” while Johnathan Livingston Seagull had over 140 rejections. And yet, these are big names today.

Remember that to editors and reviewers, you are not visible; only your writing is. They are simply doing their job of assessing that writing, just as you are doing your job of converting your thoughts into writing. You should be happy that someone is actually spending their time going through your work. Believe me, not many get even this far.

Maintaining the fine line between processing negative feedback and rejection and feeling good enough about one’s work not to give up is really tough. If we do not listen to comments or criticisms on one hand we never grow but if we take these too seriously we may become paralysed that we are no longer able to do function adequately. The hurt is more because the rejection is for words that you have conceived and put down on paper. That is why we remember the rejections more than the acceptances; we can vividly recall the scathing remarks even 15 years after the event. There are those who recommend that you keep your entire rejection letters as a mark of being battle-hardened, but that is certainly not for us. Our biggest critic is our own self! We need to be able turn off this internal critic at the critical points; off when you are writing and on when you are reading what you have written.

Not all are born with scientific writing skills. We learn as we write; and we learn by reading the famous authors. Our writing styles are defined by the choice of authors we read, so it is important that while you read famous literature, make sure to evolve your own style, something a tad different from the style of your mentor.

Publishing houses and editors are not in the habit of rejecting for the sake of rejections; they are merely trying to ensure that the highest standards of scientific writing are maintained. They try to assist authors in every possible way. It is not in their interest to stunt the growth of the writer community. It is important that if scientific writing is to progress, every writer needs to learn how to cope with rejection.

So the next time you stand before that mirror, don’t ask the obvious. Simply stare into it and accept the truth it beholds!
